# A comprehensive review on the decontamination of lead(ii) from water and wastewater by low-cost biosorbents

**DOI:** 10.1039/d2ra00796g

**Published:** 2022-04-12

**Authors:** Jonas Bayuo, Mwemezi Rwiza, Kelvin Mtei

**Affiliations:** Department of Materials Science and Engineering, The Nelson Mandela Institution of Science and Technology Postal Box 447 Arusha Tanzania bayuoj@nm-aist.ac.tz; Department of Science Education, C. K. Tedam University of Technology and Applied Sciences Postal Box 24, Navrongo, Upper East Region Ghana jbayuo@cktutas.edu.gh

## Abstract

The disadvantages of conventional methods in water and wastewater management including the demand for high energy consumption, the creation of secondary toxic sludge, and operation cost are much too high for developing countries. However, adsorption using low-cost biosorbents is the most efficient non-conventional technique for heavy metals removal. The high adsorption capacities, cost-effectiveness, and the abundance of agricultural waste materials in nature are the important parameters that explain why these biosorbents are economical for heavy metals removal. The present investigation sought to review the biosorption of lead [Pb(ii)] onto low-cost biosorbents to understand their adsorption mechanism. The review shows that biosorption using low-cost biosorbents is eco-friendly, cost-effective, and is a simple technique for water and wastewater treatment containing lead(ii) ions. The batch biosorption tests carried out in most studies show that Pb(ii) biosorption by the low-cost biosorbents is dependent on biosorption variables such as pH of the aqueous solution, contact time, biosorbent dose, Pb(ii) initial concentration, and temperature. Furthermore, batch equilibrium data have been explored in many studies by evaluating the kinetics, isothermal and thermodynamic variables. Most of the studies on the adsorptive removal of Pb(ii) were found to follow the pseudo-second kinetic and Langmuir isotherm models with the thermodynamics variables suggesting the feasibility and spontaneous nature of Pb(ii) sequestration. However, gaps exist to increase biosorption ability, economic feasibility, optimization of the biosorption system, and desorption and regeneration of the used agricultural biosorbents.

## Abstract 2

Biosorbents are found promising for the detoxification of water contaminants. This comprehensive review indicates that these biosorbents are more efficient and cost-effective for the purification of water and wastewater containing lead(ii) ions.

## Graphical abstract

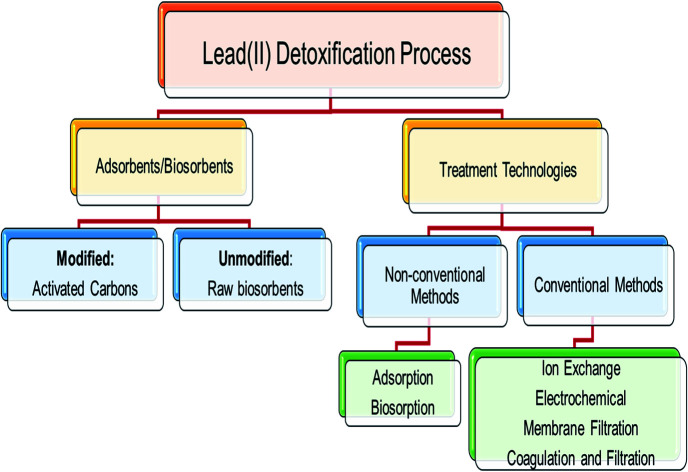

## 1. Introduction

Agricultural and industrial activities have become pollutant sources in recent times, primarily concerning the bioaccumulation of toxic metals in the ecosystem. This results in a significant escalation of the amount of these metals in surface and ground waters thereby contaminating the aquatic bodies. Heavy metals are hazardous and detrimental to living species especially when they could be distributed through the food chain.^1^ The manifestation of toxic ions in the aquatic environment is a fundamental universal concern attributable to their continuous-release, venomous nature and other deadly effects on the receiving aqueous media.

**Fig. 1:**
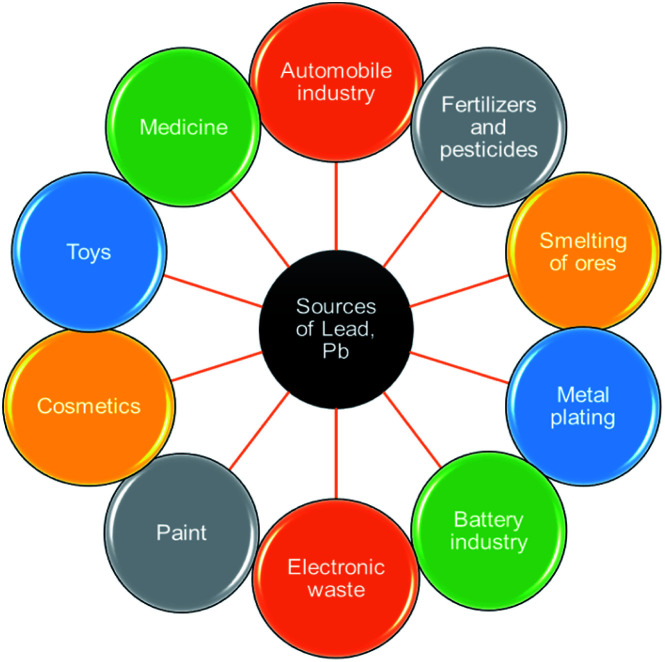
Various sources of Pb(ii) pollution in the environment (this figure has been adapted from Mohd *et al.*^7^ with permission from Springer, copyright 2021).

**Fig. 2:**
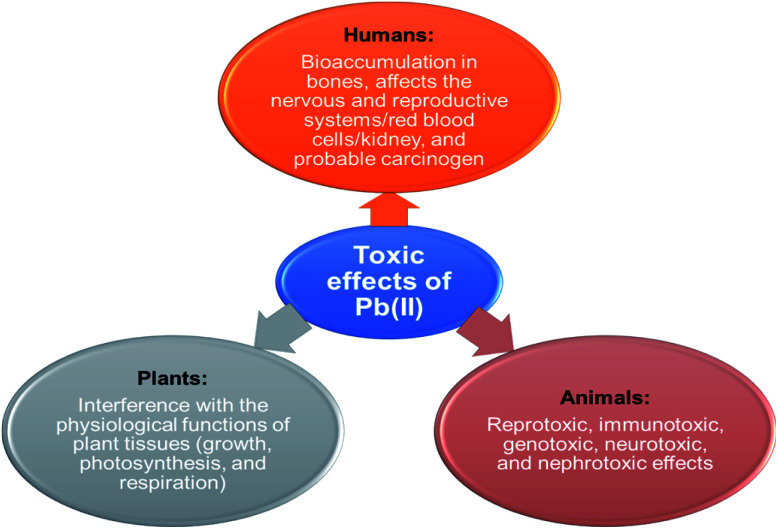
Toxic effects of Pb(ii) on plants, animals, and humans (this figure has been adapted from Abidli *et al.*^8^ with permission from Elsevier, copyright 2021).

Lead(ii) is commonly present in effluents and sewages from industries such as paint, pesticides, battery, mine, and smelting. The various sources of Pb(ii) contamination in the environment are depicted in Fig. 1. Reports indicate that grown-ups engross 5–15% of lead(ii) and nearly 5% of it is being retained and the existence of 0.5–0.8 μg per mL of lead(ii) in the blood of living organisms leads to numerous health conditions.^2^ The toxicity of lead(ii) leads to widespread health disorders including pregnancy miscarriages in women, severe stomachache, hypertension, impaired blood synthesis, brain, and kidney damage.^3^ Besides, lead(ii) is a universal pollutant on earth, and aqueous media and its exposure cause feebleness in ankles, fingers, and wrists.^4^ Some of the toxic effects of Pb(ii) on plants, animals, and humans are shown in Fig. 2.

Many treatment systems have been probed for heavy metals reduction from wastewater and water; however, many of these management processes are significantly pricy and incompetent of eliminating low-level concentrations of heavy metals. In comparison, adsorption (biosorption) appears to be more resourceful and used worldwide owing to the greater yield and cost-effectiveness of the biosorbents.^5^ Biosorption is a simple and efficient reversible reaction that takes place between heavy metals and biomass functional groups. Several studies have demonstrated Pb(ii) adsorption potentials by natural and agro-based materials leading to multiple sorption mechanisms involving ion exchange, precipitation with inorganic components, interactions with π electrons, and complexation with oxygen-containing functional groups.^6^

The objective of the present study is to conduct a review on the biosorption of lead(ii) ions from water media on low-cost biosorbents especially those obtained from natural and agro-based materials. The effects of biosorption variables as well as biosorption isotherms, kinetics, and thermodynamic variables evaluated from the adsorption equilibrium data were also reviewed.

## 2. Heavy metals

In the environment, trace metals exist naturally, particularly in the Earth's crust, and add to the balancing of the planet.^9^ At the non-anthropogenic origin, heavy metals always exist at background concentration levels where their presence in soils could be due to weathering of parent rocks. In the ecosystem, heavy metals occur naturally with large variations in concentration. Heavy metals pollution emanates from metals purification including various sources. Especially, copper smelting, nuclear fuels preparation, and electroplating of metals have led to the production of chromium and cadmium.^10^ Other sources of metal ions pollution include wet and dry fallouts of atmospheric particulate matter; dead and decomposing vegetation, animal matter, as well as from man's activities.^9,11^ Anthropogenic activities that result in toxic metal pollution are cement industry and mining operations, smelting procedures, steelworks, burning of coal and oil.^12^ Wuana and Okieimen^13^ indicated that agricultural fields could be contaminated due to improper treatments and disposal of mine drainages. Heavy metal ions can be in existence for a lengthy time in different inorganic and organic colloids before being exposed to living species.^14^ They are indestructible and do not decay with time. Most metal ions are present at trace levels in soils, vegetation, and in humans, plants, and other living species as micro-elements.^15^ Traces of heavy metals are not lethal in plants and animals at low concentration levels. However, there are few exemptions, cadmium, chromium, mercury, and lead are poisonous at very low concentrations.^16^

Toxic metals can enter the human system *via* food, air, and dermal contact absorption.^17^ They also accumulate in aquatic biota, soils, and plants.^9^ Biomagnification of metal ions may occur when an organism ejects it slower than it ingests and this can be detrimental to living organisms.^18^ The bioaccumulation of heavy metal ions tends to be very hazardous as a result of their extended biological half-lives.^19^

**Fig. 3:**
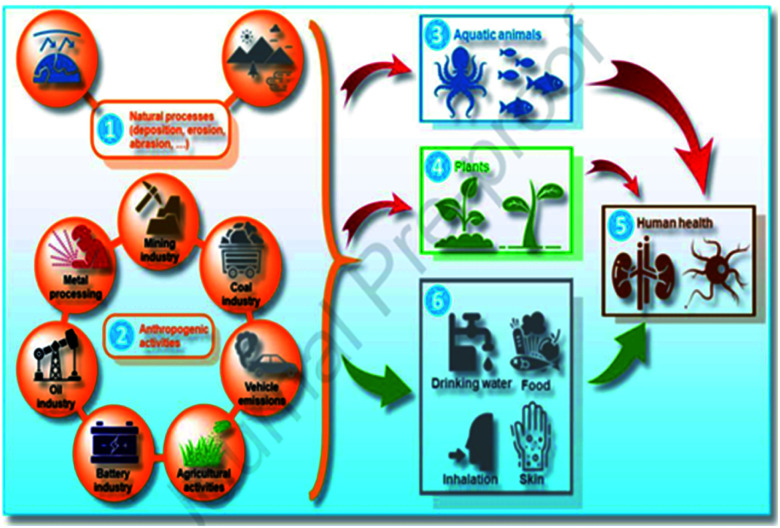
Schematic illustration of the pathway of heavy metals and metalloids pollutants in the ecosystem (this figure has been adapted from Abidli *et al.*^8^ with permission from Elsevier, copyright 2021).

As shown in Fig. 3, heavy metals can pollute the ecosystem due to various pathways through natural processes [1], and anthropogenic activities [2], leading to detrimental effects in the environment including direct pollution of the aquatic environment [3], plants [4], as well as triggering toxic effects on humans [5] through different intake paths such as food chain (drinking water and food), inhalation, and skin contact [6].

### 2.1 Heavy metals presence and pollution in the ecosystem

The discharge of metal ions into the ecosystem occurs over wide-ranging processes and pathways including to the surface waters *via* runoff, transport to soil; hence, into groundwaters and crops as well as to air during combustion.^20,21^ Heavy metal ions occurrence in the soils could be accredited to different environmental factors, including, but not restricted to soil characteristics, parent materials, and human activities for example farming and irrigation.^22^ Toxic metals in soil can influence crop growth negatively and as well as interferes with the degeneration of major cell organelles, metabolic processes in plants, inhibition of respiration and photosynthesis.^23^

Furthermore, the production and usage of synthetic products such as paints, pesticides, and batteries can lead to heavy metal pollution of agricultural soils and the environment at large.^24^ Contamination of soils by heavy metals is primarily due to the use of wastewaters obtained from mining operations to irrigate paddy fields.^25^ Heavy metals have the potential of polluting groundwater because of their high rate of transfer in soil profiles. The potentiality of metal ions accumulation and bioaccumulation in soils leads to serious food chain contamination.^26^ In Ghana, organochlorine pesticides are widely been used for public health and agricultural purposes for more than 40 years with their remains found in sediments, water, humans, and crops.^27^ For instance, crop and vegetable farming had led to misuse and excessive use of pesticides. In Ghana, pesticides form part of agriculture and it has been estimated that about 87.00% of farmers depend on pesticides to prevent diseases and pests on vegetable farms, rice fields, cotton, and cocoa plantations and among others.^28^ It has also been reported that several toxic metals are found to be present in drinking water media in some mining areas in the country.

Global worries have suddenly increased over toxic metals discharged from various sources into the environment due to their inability to degrade and so they linger in the environment.^22^ Metal circulation between soil and vegetation is a very important issue in evaluating the effect of metals on the environment.^29^

**Fig. 4:**
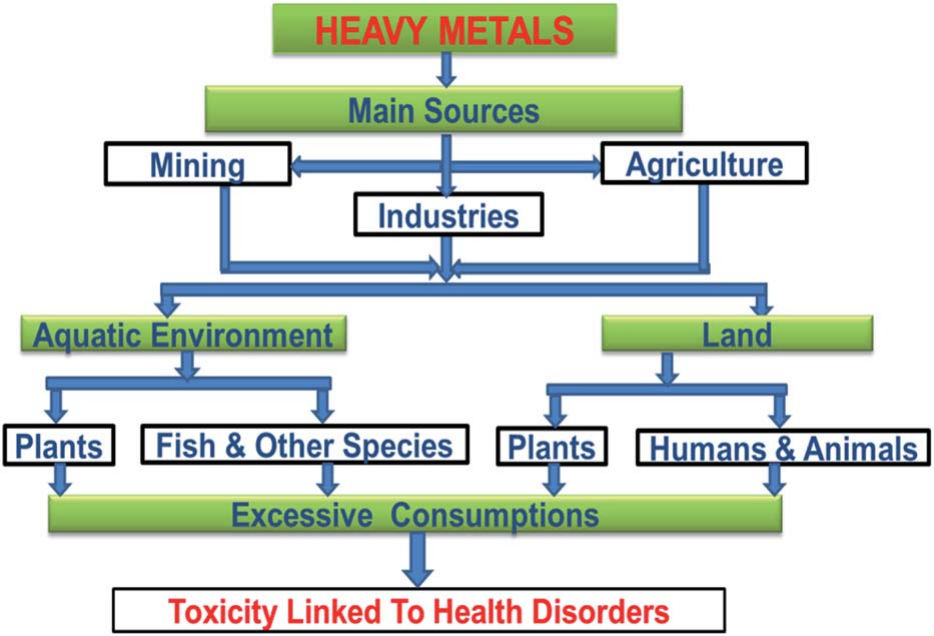
Sources of heavy metals and their impact on living organisms.

The main sources of heavy metals in the environment from anthropogenic activities and their implication on human beings and other life forms are presented in Fig. 4.

### 2.2 Effect of heavy metals

**Fig. 5:**
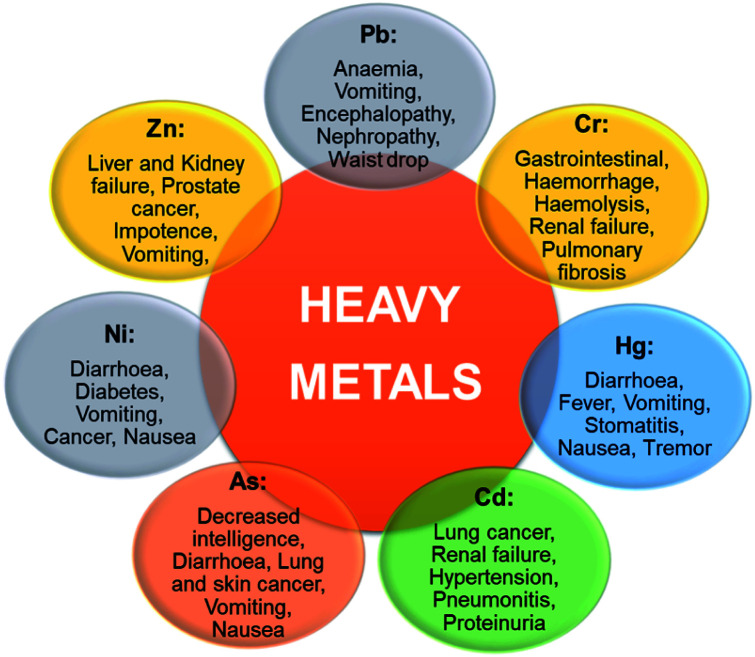
The effects of heavy metals on human health (this figure has been adapted from Mohd *et al.*^7^ with permission from Springer, copyright 2021).

**Fig. 6:**
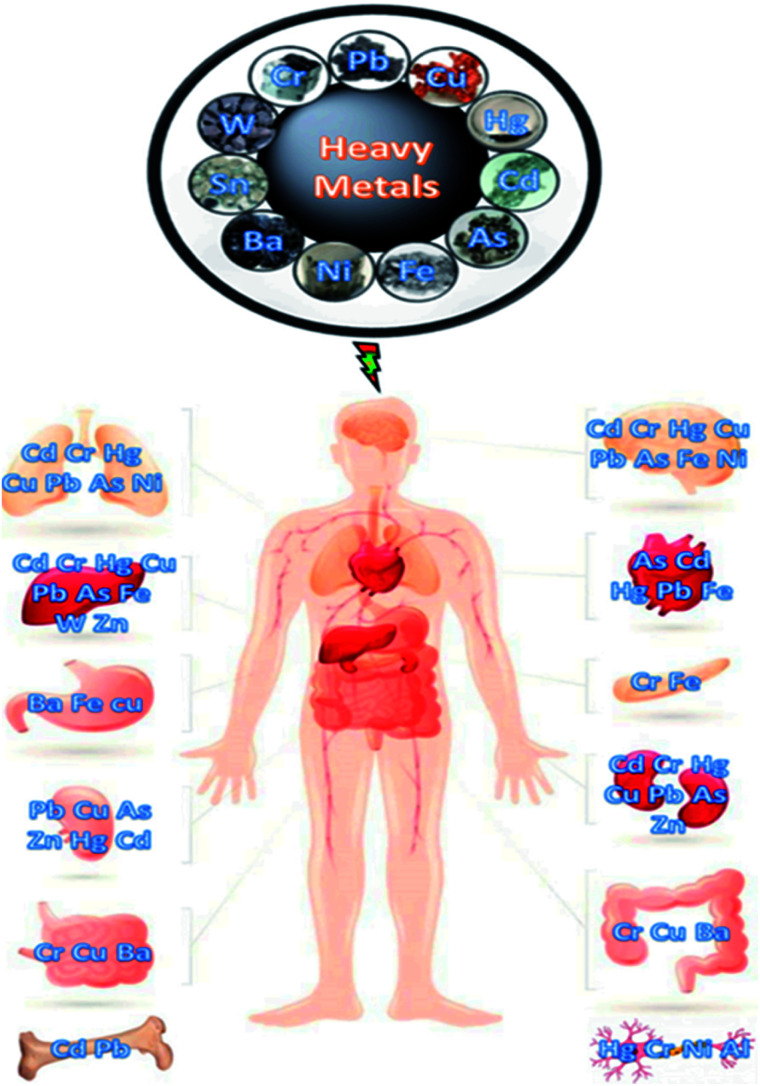
Illustration of human organs affected due to heavy metals toxicity (this figure has been reproduced from Perumal *et al.*^40^ with permission from MDPI, copyright 2021).

The toxicity of metal is mainly defined based on its concentration required to cause an acute response generally a sub-lethal response or death. Several trace elements are recognized to be noxious to human beings and other living species when consumed beyond their concentration required for healthy growth. Some of these heavy metals [arsenic (As), chromium (Cr), cadmium (Cd), lead (Pb), nickel (Ni), mercury (Hg), and zinc (Zn)] and their harmful effects in living organisms when bio-accumulated in higher concentration than their acceptable levels are presented in Fig. 5. There could be a high tendency of metal ions concentration build-up in living tissues once they become part of the food chain and cause severe health disorders when ingestion exceeds the permitted concentration.^30^ Heavy metal toxicity has been found to have an inhibitory influence on enzymatic functions, plant development, stoma roles, photosynthesis processes as well as the destruction of the root system.^31^ The health risks assessment of metal ions pollution of potable water bodies are most imperative due to their lethal effects and capability of bioaccumulation *via* the food chain.^32^ Different metals show alterations in the level of harmfulness, some are carcinogenic though other effects may include cardiovascular,^33^ epidermal systems alteration,^34^ and hematological, neurological, gastrointestinal, immunological, and musculoskeletal effect.^35^Fig. 6 depicts the human organs usually affected as a result of the discharge of excessive heavy metal ions into the environment from different sources. Highly toxic metal ions cause destructive effects even at small concentration levels.^36^ The dangers of heavy metals have become the most current environmental health concern because they are potentially dangerous due to bioaccumulation through the food chain.^25^ Mostly, heavy metals are distributed, concentrated, and chemically modified through human activities that may increase their toxicity. These activities result in greater concentrations of metal ions relative to their normal background concentrations.^25^ Contamination with metal ions poses a serious threat to agriculture and other sources of food for humanity, reducing plant growth and reducing the resistance to pests and diseases. This affects the quality of groundwater, plant growth, food, and microbes.^10^ The effects of heavy metals on microbes can lead to reduced waste degradation and nitrogen fixation, inefficient nutrient circulation, and impaired enzyme synthesis.^25^ Poisonous ions persist in the ecosystem for a prolonged time and do not decompose.^37^ Rapid exposure to toxic trace elements in marine and terrestrial organisms can have a negative toxic effect.^38^ For example, coastal fish and seabirds are often observed for the presence of toxins, such as exposure to poisonous metals due to the food chain. It is generally difficult to predict heavy metals exposure effects on species since these metals may be essential or non-essential and very low concentrations of essential metals can be as harmful as high concentrations.

Table 1 depicts the Maximum Allowable Limits (MAL) and health implications of different heavy metals.^22,39^

**Table 1:** Maximum allowable limits (MAL) and health implications of heavy metals

Heavy metal	MAL for effluent discharge from industries (mg L^−1^)		MAL for drinking water (mg L^−1^)			Health implication
	WHO					
	Inland surface water bodies	Marine coastal regions	WHO	European Union standard	USEPA	
Arsenic	—	0.2	0.01	0.01	0.01	Cancerous and gastrointestinal disorder
Cadmium	0.1	2.0	0.003	0.005	0.005	Causes cancer, dyspnoea, and lung fibrosis
Copper	0.05–1.5	3.0	2.0	0.2	1.3	Causes stomach pain, irritation of the eyes, nasal cavity, mouth, and headache
Chromium	—	2.0	0.05	0.05	0.1	Causes tumor in lungs and cancer
Iron	0.1–1.0	3.0	0.2	0.2	0.3	Causes hypertension, contraction of the blood vessel and pulse rate
Lead	0.1	2.0	0.01	0.01	0.15	Causes anemia, joint and muscle ache, elevated blood pressure, kidney inflammation, and carcinogen
Manganese	0.05–0.5	2.0	0.5	0.05	0.05	Fatigue, blindness, and sexual impotence
Mercury	—	0.01	0.001	0.001	0.02	Diarrhea, headache, abdominal effects, loss of appetite, and paralysis
Nickel	—	5.0	0.02	0.02	0.1	Cancer of the lung and acute bronchitis
Zinc	5.0–15.0	15.0	3.0	—	5.0	Causes discomfort and metal fume fever

### 2.3 Application of conventional adsorbents in heavy metals decontamination

Earlier adsorbents used for metal ions removal were commercial adsorbents and have been used globally and extensively for controlling water contamination. In literature, the commonly utilized conventional adsorbents are activated carbons, ion-exchange resins (polymeric organic resins), and inorganic materials such as activated alumina, silica gel, zeolites, and molecular sieves. However, only four types of generic adsorbents have enormous surface areas and have dominated the commercial use of adsorption: activated carbons > zeolites > silica gel > activated aluminas.^41^

Silica gel is worldwide recognized as an inorganic polymer that is used in chromatographic columns. Its high porous surface topology and enormous specific surface area are key for effective heavy metal ion interaction. In furtherance, silica gel is easily available as a substrate for immobilizing various chemical functional groups.^42^ Several silicate-derived gels have been explored to extract, stabilize, and/or decontaminate arsenic and other heavy metal ions from water and wastewater.^43^ Due to its strong physical strength, endurance, and high chemical stability, silica gel has been utilized in the sequestration of certain contaminants including solid wastes.^43,44^ El-Moselhy *et al.*^44^ were successful in developing hydrated iron oxide nanoparticles using silicate matrix, resulting in high arsenic detoxification. Mesoporous silica materials of MCM-41 type were efficiently utilized by Zhu *et al.*^45^ in the removal of Cu(ii), Pb(ii), and Cd(ii) from aqueous solutions. More so, a silica gel material modified with nitrilotriacetic acid (NTA-silica gel) was applied for the elimination of Cu(ii), Cd(ii), and Pb(ii) from the aqueous system, which indicates high removal efficiency of the three heavy metal ions.^42^ Furthermore, silica gel polymer was proven to be effective in Ni(ii) removal from aqueous solution.^46^

Zeolites are micro-porous crystal-like solids with definite structures and are naturally-occurring materials comprising hydrated aluminosilicate with maximum cation exchangeability with metal ions.^47^ Zeolite has a highly porous structure in three-dimensional crystal lattices that display strong cation interaction and ion exchange ability with heavy metal ions.^48^ Due to their high porosity and sieving characteristics, zeolites are a good choice for removing heavy metal ions from untreated wastewater. They also have a high ion exchange ability, and the exchangeable ions (Na^+^, Ca^2+^, and K^+^ ions) are largely harmless.^49^ Based on their quick advancements in properties, zeolites are gaining in popularity and are being used in a variety of innovative applications. Biomedical, radioprotection, wastewater purification, biosensor, pure, and applied chemical applications are all driving interest in them.

When using zeolites in sorption, the surface area is not a matter of concern because it is a selective process and it is reversible.^50^ Zeolites are used practically in the fuel industries as water softeners, manufacture of detergents, catalysts, and making molecular sieves. Quite a lot of zeolites have been employed as adsorbents for the treatment of contaminants in water and wastewater. A zeolite produced from fly ash was capable of decontaminating Hg(ii) and Pb(ii) ions from aqueous media.^51^ Chen *et al.*^52^ designed and prepared zeolite cotton as a form of filter for household water treatment. More so, zeolite composite adsorbents were applied to simultaneously detoxicate heavy metals and total coliforms from wastewater.^53^ A high-quality zeolite type A was produced and utilized successfully in removing heavy metals from water systems.^54^ Besides, zeolite-alginate composites were found to be promising in the Pb(ii) removal from contaminated water solutions.^55^ Makki^56^ employed zeolite A4 for Cd(ii) and Pb(ii) decontamination in water. Also, Cu(ii), Ni(ii), and Pb(ii) ions in an acid mine drainage were removed using Philippine natural zeolite.^57^

Activated alumina is one of the most extensively utilized adsorbents for heavy metals sequestration due to its high affinity for metal ions.^58^ It consists of porous aluminum oxide with a wide surface area that is resilient to thermal stress and abrasion. It does not swell, shrink, soften, or disintegrate throughout the adsorption process.^59^ Furthermore, activated alumina has outstanding adsorption capabilities and a high ion uptake capacity, as well as being cost-effective, safe, and ecologically friendly, making it suitable for use in wastewater treatment.^60^ Activated alumina has a good surface area and is frequently applied to get rid of oxygenates and mercaptans of hydrocarbons and especially, fluorides in water.^61^ A nano-alumina was produced and used effectively in As(v) decontamination from an aqueous solution.^62^ Furthermore, novel γ-alumina nanoparticles were developed for Ni(ii) decontamination from the solvent phase and found proficient.^63^ In a study, a mesoporous carbon stabilized alumina was employed in removing Cd(ii) and Pb(ii) ions from aqueous media.^64^ Besides, an alumina composite was shown to be effective in the removal of both Cr(vi) and methylene blue from aqueous solutions.^65^

Globally, the elimination of noxious pollutants in water has been attributed to activated carbons. The well-known and lasting adsorbent in water and wastewater treatment is charcoal. The preparation of activated charcoal involves three stages; dehydration, carbonization, and activation. Due to the high surface area of activated carbons, they are highly recognized and recommended as proficient adsorbents for water contaminants treatment and gas purification.^66,67^ Activated carbons are black and made up of solid carbonaceous materials with high porosity, internal surface area, and mechanical strength. Granular activated carbon, powdered activated carbon, and activated carbon cloth are among the different forms of activated carbon used to remove contaminants from water and wastewater.^68^ The constant utilization of activated carbons has produced fruitful outcomes in contaminant management processes.^41^ Hu *et al.*^69^ report the applicability of humic acid-impregnated activated carbon in removing Cu(ii). Meanwhile, Jjagwe *et al.*^70^ previously reviewed the synthesis and application of granular activated carbon from biomass waste materials for water treatment. Besides, Manjuladevi *et al.*^71^ investigated Cr(vi), Ni(ii), Cd(ii), and Pb(ii) ions removal from aqueous solutions using activated carbon prepared from *Cucumis melo* peel. Fan and Anderson^72^ carried out a related study using granulated activated carbon to eradicate Cu(ii) and Cd(ii). Also, activated carbon has been applied in the decontamination of Pb(ii) as recounted by many studies.^73,74^

Regardless of the capabilities and prolific application of commercial activated carbons, there are some constraints on usage attributable to its high cost. For that reason, attention has been directed towards the preparation and production of cost-effective adsorbents from natural materials that are readily available, carbonaceous, and easy to activate.^75^ To produce activated carbon, an organic material undergoes thermal degradation for subsequent decomposition into carbon granules.^70^

### 2.4 Application of agricultural waste materials in heavy metals removal

**Fig. 7:**
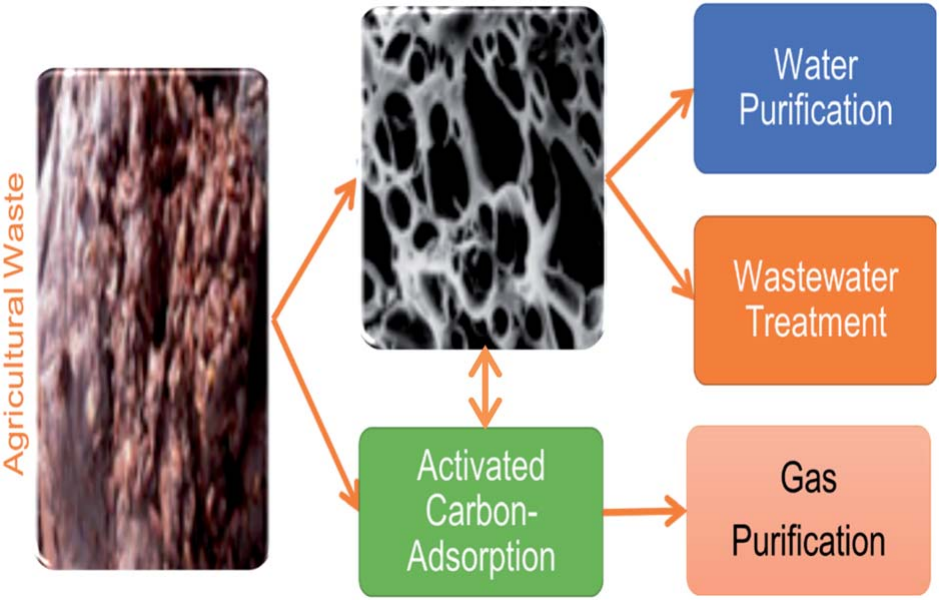
Application of low-cost activated carbons derived from agricultural wastes for environmental remediation (this figure has been adapted from Heidarinejad *et al.*^85^ with permission from Springer, copyright 2020).

Commercially activated carbons are expensive adsorbents regardless of their widespread use in water and wastewater treatments. This has generated an increased interest in the production of safe, cheap, and economical adsorbents, which are more economical than commercially available activated carbon aimed at removing heavy metals from polluted resources.^76,77^ Natural and waste materials of agriculture and industries are extensively being modified and converted into activated carbons aimed at heavy metals cleansing from wastewater.^75^ For instance, Fig. 7 demonstrates the application of activated carbons as adsorbents derived from agricultural wastes for water and wastewater treatment. An effective adsorbent should have a wide adsorption capability, a fast rate of sorption, be easy to regenerate or retrieve from water, have high porosity, and have a small pore diameter, because adsorption ability is proportional to the amount of surface area available.^78^ Typical adsorption properties of these materials are derived polymers complexes such as lignin, simple sugars, hemicellulose, proteins, lipids, and starch with diverse functional groups. These constituents play a vital role in the adsorption of heavy metal ions as they can form complexes and chelates with heavy metal ions. They are capable of replacing hydrogen ions with hazardous heavy metal ions dissolved in the aqueous media or donating a pair of electrons to bind these metal ions.^79^

The functional groups that are present in agricultural wastes include carbonyl, acetamido, phenolic, amido, carboxyl, amino, alcohols, esters, and sulphydryl.^80^ It is these groups, which possess an affinity for complexation with metals. During adsorption studies, various studies have confirmed the existence of functional groups and the binding ability of these groups with metal ions through spectroscopy analysis.^81–83^

**Fig. 8:**
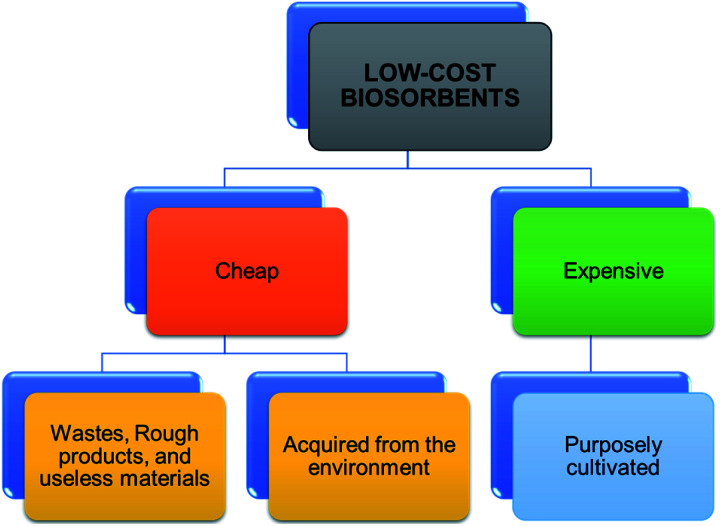
Sources of agro-based biosorbents.

**Fig. 9:**
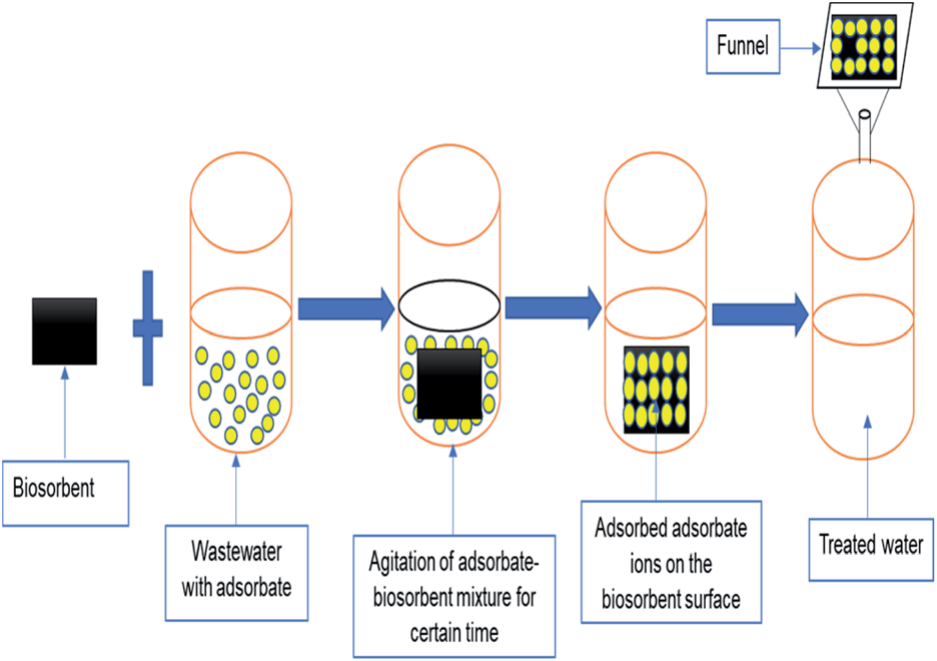
Adsorption process to clean up polluted water using low-cost adsorbents.

Toxic metals elimination by dint of low-cost biosorbents is regarded as more promising and cost-effective since there are numerous natural resources available locally and plentifully, which could be used as low-cost biosorbents.^84^ The development of agro-based materials into less expensive biosorbents is well acknowledged as a prospective and economical for the treatments of pollutants in water. The sources of agro-based biosorbents are presented in Fig. 8. The increasing number of studies or researches and publications on the adsorption process (as shown in Fig. 9) using low-cost adsorbents conclude and recommend the increasing interest in the search for more low-cost adsorbents that are suitable for removing pollutants. As a result, several studies have reported the uses of agro-based adsorbents in eliminating heavy metals from water, wastewater, or effluents. Some of these studies are summarized in Table 2.

**Table 2:** Agricultural waste bioadsorbents for heavy metal ions sequestration

S/N	Bioadsorbent	Metal ion	Removal efficiency or *Q*_max_	References
1	Thiolated saw dust	Pb(ii) and Co(ii)	2.87 mg g^−1^	84
2	Groundnut shell	Cr(vi), Pb(ii)	87.6 and 96.61%, respectively	82 and 86
3	Shea fruit biomass	Cd(ii)	76.86%	76
4	Coffee waste-derived biochars	Cd(ii) and Pb(ii)	11.41 and 1.18 mg g^−1^, respectively	122
5	Banana peels	Cu(ii) and Pb(ii)	99.79 and 88.94%, respectively	123
6	Spent tea leaves	As(v)	87%	124
7	Coconut shell char	Ni(ii)	0.58 mg g^−1^	125
8	Olive stones	Cd(ii), Cu(ii), Pb(ii) and Cr(vi)	77.4, 80.5, and 94.5%, respectively	79
9	Brewed tea waste	Pb(ii), Zn(ii), Ni(ii), and Cd(ii)	1.197, 1.457, 1.163 and, 2.468 mg g^−1^, respectively	126
10	Orange peel	Cd(ii)	128.23 mg g^−1^	127
11	Jute stick activated carbon	Cd(ii)	73.53 mg g^−1^	128
12	Oil palm fibers	Pb(ii), Cu(ii), Fe(ii) and Zn(ii)	16.67, 16.59, 16.65 and 16.54 mg g^−1^, respectively	129
13	Sunflower waste carbon	Cd(ii)	99.8%	130
14	Modified corn stalks biochar	Cr(vi)	138.89 mg g^−1^	131
15	*Dialium guineense* seed shell	As(iii)	47.08 mg g^−1^	132
16	Sagwan sawdust	Cr(vi)	9.62 mg g^−1^	133
17	*Saccharomyces cerevisiae* biomass	As(iii) and As(v)	66.2 and 15.8%, respectively	134
18	Activated carbon from sugarcane bagasse	Hg(ii)	61%	135
19	Succinylated hay	Cd(ii) and Ni(ii)	75.19 mg g^−1^ and 57.77 mg g^−1^, respectively	136
20	Carbon derived from corn straw	Cr(v)	175.44 mg g^−1^	137
21	Modified chicken feather	88.9%	88.9%	138
22	*Albizia lebbeck* pods	Pb(ii), Cd(ii), Zn(ii) and Cu(ii) ions	7.17, 7.81, 8.26, and 8.68 mg g^−1^, respectively	88
23	Fe-modified rice straw biochars	As(v)	69.6%	88
24	Spent tea leaves	Cr(iii)	95.42%	139
25	Bagasse biochar	Pb(ii)	75.376%	91
26	Corn and rice husks	Pb(ii)	>90%	140
27	Rice husk ash	Pb(ii)	75%	141
28	Corn silk	Cu(ii) and Zn(ii)	15.35 and 13.98 mg g^−1^, respectively	142
29	Mung bean husk	As(v)	98.75%	143
30	*Typha angustifolia* and *Salix matsudana* branches	Cd(ii) and Pb(ii)	90%	144
31	Mustard waste biomass	Pb(ii), Zn(ii), and Cd(ii)	94.56, 96.15 and 76.48%, respectively	145
32	Banana peel	Cd(ii) and Pb(ii)	93.2 and 83.78%, respectively	146
33	Coco-peat biomass	Pb(ii), Cd(ii), Cu(ii), and Ni(ii)	0.484, 0.151, 0.383 and, 0.181 mmol g^−1^, respectively	147
34	Coffee residues	Pb(ii) and Zn(ii)	96 and 44%, respectively	148

### 2.5 Elimination of Pb(ii) ions using low-cost adsorbents

Bayuo *et al.*^86^ studied the optimization of adsorption factors to eliminate lead(ii) from an aqueous medium by groundnut husk. The influences of three factors (contact time, pH, and initial metal Pb(ii) concentration) on two responses (removal efficiency and adsorption capacity) were explored by Central Composite Design. The optimal operative conditions for Pb(ii) removal were established at 90 min contact time, pH of 8 and 75 mg L^−1^ Pb(ii) initial concentration by a 0.966 desirability. At this operating condition, the optimal percent removal of Pb(ii) and adsorption capacities were 90.26% and 3.428 mg g^−1^ respectively. The equilibrium data agreed with the Langmuir isotherm and pseudo-second-order kinetic model.

Hydroxyapatite nanostructures were synthesized by microwave irradiation from eggshells by Safatian *et al.*^87^ This nano adsorbent was applied to eliminate lead(ii) from wastewater. The impacts of solution pH, contact time, dosage, Pb(ii) initial concentration, and temperature on the elimination of lead(ii) were examined revealing Pb(ii) depollution dependence on these parameters. Langmuir and Freundlich's models were tried and the results show good fitness to Langmuir isotherm. The synthesized nano adsorbent displays great adsorption abilities for removing lead(ii) from water media.

Mustapha *et al.*^88^ produced a biosorbent using *Albizia lebbeck* pods to remove Pb(ii), Cd(ii), Zn(ii), and Cu(ii) ions from aqueous media. The influences of many parameters including solution pH, contact time, dosage, initial concentration, and temperature were studied by batch mode. Equilibrium and kinetic experiments were conducted as well as change in enthalpy entropy and free energy was determined. The study confirmed that *Albizia lebbeck* pods are capable of removing metals from the aqueous environment. The experimental data fitted the Langmuir model with a high correlation coefficient (*R*^2^ > 0.94); however, the kinetic data obeyed the pseudo-second-order model. The adsorption thermodynamic variables attested that the biosorption of these metals was endothermic, non-spontaneous, and spontaneous at lower and higher temperatures respectively.

Boulaiche *et al.*^89^ reported the use of *Posidonia oceanica* in removing Pb(ii), Cd(ii), Ni(ii), Cu(ii), and Zn(ii) using the batch procedure. The influence of pH, contact time, initial metal ion concentration, and the temperature was considered. Sorption kinetics and isotherm models were verified for the biosorption of the metals. The concentrations of the metals adsorbed were found to be 48.33, 43.9, 41.02, 37.90, and 30.22 mg g^−1^ for Pb(ii), Cu(ii), Ni(ii), Zn(ii), and Cd(ii), respectively. The biosorption kinetic data conformed to the pseudo-second-order model while the isotherm equilibrium data rightly fitted Langmuir models for all the metals investigated. The biosorption of Pb(ii) was endothermic and spontaneous as suggested by the thermodynamic variables evaluated.

A novel natural adsorbent obtained from thorns was utilized by Alatabe and Kariem^90^ to remove lead(ii) from wastewater. The influence of diverse factors (pH, contact time, initial concentration, and different electrolytes) was investigated by batch technique to optimize conditions for which optimal biosorption can be achieved. The experimental data were scrutinized with isotherm and kinetic equations. The study revealed that the adsorbent has a high ability to eliminate Pb(ii) and at a pH of 6, optimum biosorption was accomplished. The removal concentration was dependent on the time of equilibrium. At higher temperatures of the isotherm studies, Langmuir model was obeyed. The Pb(ii) ions adsorption was influenced by raising electrolyte concentrations. According to the experimental data, the pseudo-second-order model illustrates better applicability indicating chemical sorption. The thermodynamic data point out Pb(ii) depollution to be endothermic and spontaneous.

Manjuladevi *et al.*^71^ examined Pb(ii), Ni(ii), Cr(vi), and Cd(ii) eradication from aqueous media using *Cucumis melo* peel-activated carbon (CMAC). The biosorption characteristics of the ions on CMAC were evaluated utilizing kinetic models. The biosorption kinetic data showed a better fit to the pseudo-first-order than the pseudo-second-order model. The optimum removal efficiencies of the ions by the CMAC were 98.78, 98.55, 97.95, and 97.96% for Ni(ii), Pb(ii), Cr(vi), and Cd(ii), respectively at 250 mg dosage of CMAC. The maximum values for the parameters for which optimum removal was achieved at 250 mg dosage, 250 mg L^−1^ for all metals, pH of 6.0 for Ni(ii), Cd(ii), and Pb(ii), and pH of 3.0 for Cr(vi) using 180 min contact time.

Bagasse is agro-based biomass that was employed by Poonam *et al.*^91^ for investigating its possibility in removing lead(ii) from effluents of the battery industry. Kinetics and batch experiments comprising several factors (pH, contact time, and dosage) were investigated using biochar produced from the bagasse waste. The maximum removal was attained at a pH of 5, 140 min contact time, and room temperature (25 ± 3 °C) using 5 g biochar. At the optimum pH of 5, the optimum percent removal and the amount of Pb(ii) adsorbed were 75.38% and 12.741 mg g^−1^ correspondingly. The equilibrium data were well represented by Langmuir suggesting monolayer and homogenous biosorption of Pb(ii). The pseudo-second-order model indicates conformity to the experimental data implying chemisorption.

Mahdi *et al.*^92^ studied lead(ii) removal from aqueous media onto biochars obtained from the biomass of date seed. The impacts of solution pH, contact time, and initial Pb(ii) concentration were conducted. Aqueous solution pH exhibited an influence on the biosorption capability of the biochar and maximum removal occurred at a pH of 6.0. The equilibrium data were effectively described by both Freundlich and Langmuir isotherms. The experimental data conformed well to the pseudo-second-order kinetic model. The study indicated that date seed-based biochar has high removal potential for Pb(ii) in comparison to other plant-derived biochars.

Gaur *et al.*^93^ examined the ability of soya bean as a biosorbent in the purification of lead (Pb) and arsenic (As) in wastewater. Batch tests were conducted and the influence of some biosorption parameters (pH, contact time, adsorbent dose, and temperature) was determined. It was found that the maximum elimination of Pb(ii) and As(iii) was achieved at 37 °C and 3.0 g per 100 mL. At a pH of 2.0 and 4.0 ± 0.26, maximum decontamination of As(iii) and Pb(ii) were attained respectively at a shaking time of 60 min. Langmuir and Freundlich's models were tested and Langmuir suitably fit equilibrium data. Thermodynamics studies suggest the biosorption of Pb(ii) using the soya bean biosorbent was exothermic.

Ferreira *et al.*^94^ studied the application of a modified cashew nutshell (*Anacardium occidentale*) (CNS) in the depollution of Pb(ii), Cd(ii), and Cr(iii) from an aqueous environment. Batch tests were conducted to determine the optimal conditions of adsorption parameters. The cashew nutshell displayed high decontamination of Pb(ii), Cd(ii), and Cr(iii). The study discovered that the maximum biosorption took place at a pH of 5.0, 4 g of the biosorbent, and a contact time of 40 min. The equilibrium data demonstrated a good agreement with Langmuir and Freundlich isotherms suggestive of the biosorption taking place on mono and multilayers.

Klapiszewski *et al.*^95^ combined lignin and silica to produce a multifunctional adsorbent with substantial sorption abilities. Batch experiments were conducted using silica/lignin hybrid in eliminating lead(ii) from water media. It was found that the efficiency of the biosorption rest on removal time, pH, and adsorbent dosage with a maximum adsorption capacity of 89.02 mg g^−1^. The experimental data of lead(ii) obtained by SiO_2_/lignin biosorbent show the best fit for Langmuir and pseudo-second-order models.

Ghogomu *et al.*^96^ utilized activated carbons produced from *Raphia hookeri* fruit epicarps using ZnCl_2_ (CAZn), H_3_PO_4_ (CAH), and KOH (CAK) as activation agents to remove lead(ii) from wastewater. Batch tests were performed to determine the impacts of contact time, solution pH, dosage, and initial Pb(ii) concentration at 27 °C. The results disclosed that optimum removal of Pb(ii) occurred at 80 min contact time for CAZn and CAH and 100 min for CAK, pH of 6 for all samples with biosorption capacities of 66.37 mg g^−1^ for CAZn, 56.30 mg g^−1^ for CAH and 28.0 mg g^−1^ for CAK, respectively. The experimental data illustrate a good fit using the Langmuir model with monolayer biosorption capacities of 72.62, 58.62, and 12.16 mg g^−1^ for CAZn, CAH, and CAK correspondingly. The kinetic investigations have shown that the biosorption system fits the pseudo-second-order model. *Raphia hookeri* based activated carbons (CAZn and CAH) were revealed to be capable of removing lead(ii) from an aqueous environment.

Nnaji and Emefu^97^ conducted experiments to investigate lead(ii) decontamination using different mesh sizes of activated sawdust of two species of timber. The best particle sizes were 0.85 and 1.18 mm for *Khaya ivorensis* and *Pycanthus angolensis*, respectively. The equilibrium data were applied to isotherm and kinetic models. The biosorption of lead(ii) using *Khaya ivorensis* and *Pycanthus angolensis* followed Langmuir and Freundlich models while experimental data conformed to the pseudo-first-order model.

Nordine *et al.*^98^ studied lead(ii) elimination from synthetic wastewater using natural materials such as pine, beech, and fir sawdust. Several biosorption variables (pH, sawdust dosage, contact time, initial Pb(ii) concentration, stirring speed, and temperature) were investigated. The study established that the pine sawdust show higher lead(ii) removal (15.5 mg g^−1^) at pH of 5.45 ± 0.05, 10 g L^−1^ of pine sawdust, initial Pb(ii) concentration of 100 mg L^−1^, and 23 ± 2 °C temperature. The Langmuir model showed a good representation of the experimental data describing the Pb(ii) biosorption mechanism as a monolayer.

Yahaya and Akinlabi^99^ investigated cocoa pod husk in lead(ii) decontamination. The adsorbent was activated chemically by treating it with dilute HNO_3_ for 24 hours. The adsorbent was modified with 7% (v/v) sulphuric acid (H_2_SO_4_) for 90 minutes. An additional modification of the adsorbent was done using 0.3 dm^3^ of pyridine and 5.67 g EDTA under reflux for 24 hours. At the maximum time of 50 min, the removal percentages of lead were 94.6% and 98.2%, respectively for the original and improved cocoa pod husk. The best-fitted model was pseudo-second-order with a high correlation coefficient ranging from 0.9776 to 1.0000. From the thermodynamic studies, the adsorption process was feasible, increase in randomness and endothermic.

Activated carbon produced using sugarcane bagasse (SCBA) and its possibility to adsorb lead(ii) from synthetic water was studied by Kane *et al.*^100^ Batch mode trials were conducted to probe the impacts of pH and dosage of SCBA on the biosorption of Pb(ii). The batch results demonstrated Pb(ii) biosorption dependence on the pH and SCBA dosage. The optimum depollution of Pb(ii) was obtained at pH 5.0 and 10 g L^−1^ SCBA dose with a removal efficiency of 87.3%. The isotherm data indicated good agreement with the Langmuir model and the maximal biosorption capacity determined from the model was 23.4 mg g^−1^.

Khandanlou *et al.*^5^ prepared nanocomposites (RS/Fe_3_O_4_-NCs) using rice straw to eliminate Pb(ii) and Cu(ii) from water. The influences of three parameters, removal time, adsorbent dose, and initial ion concentration were examined on Pb(ii) and Cu(ii) removal possibilities. The optimum removal conditions of Pb(ii) and Cu(ii) were found at removal times of 41.96 and 59.35 min; initial ion concentrations of 100 and 60 mg L^−1^ and 0.13 g of the biosorbent for both ions, respectively. The optimum percent removal of Pb(ii) and Cu(ii) was attained at 96.25% and 75.54%, respectively. The isotherm and kinetic data obeyed the Langmuir isotherm and pseudo-second-order models for both ions.

Shi *et al.*^101^ investigated heavy metals [Pb(ii), Co(ii), and Cu(ii)] elimination in water media using arborvitae leaves. Single and competitive component absorption experiments were performed and the adsorption capability of the leaf was found to be dependent on the pore diameter and surface. The optimal uptake capacities of Pb(ii), Co(ii), and Cu(ii) at an equilibrium time of 120 min were 7.64, 1.84, and 3.38 mg g^−1^, respectively. The single-component adsorption showed that the decontamination of the ions depended on increasing order of electronegativity, Pb (2.33) > Cu (1.90) > Co (1.88). Conversely, the ternary component biosorption process revealed that the uptake of Pb(ii), Co(ii), and Cu(ii) ions onto the biosorbent declined from 35.84 to 9.32 mg g^−1^, 6.78 to 1.54 mg g^−1^, and 7.94 to 3.07 mg g^−1^, respectively. At pH 5.5, maximum decontamination of the ions was achieved and the equilibrium data correlated well with the Langmuir model.

Paliulis^102^ conducted a study using peat to remove Pb(ii) from aqueous solutions. The study revealed an increase in the uptake capability of peat with the rising contact time, initial concentration of Pb(ii) and was remarkably affected at a pH range of 4.0–7.0. At pH of 6.0, a contact time of 360 min and Pb(ii) initial concentration of 100 mg L^−1^, maximum Pb(ii) biosorption (9.489 mg g^−1^) was achieved by the peat. The Langmuir and Freundlich isotherms were employed in the analysis of the biosorption properties of Pb(ii) with Freundlich being the best fit model.

Misihairabgwi *et al.*^103^ studied heavy metals decontamination from water media utilizing activated carbon obtained from agroforestry wastes including rice husk, baobab shells, pigeon pea husk, macadamia nut shells, marula stones, and *Moringa oleifera* husks. The uptake capacity was increased when the pH was raised from 4.0 to 6.0 but at a pH of 6.0, 60% uptake of Hg(ii) was achieved for all the activated carbons. The elimination of Pb(ii) was observed to increase by 22% in removal efficiency in comparison to activated carbons of *Moringa oleifera* husks, pigeon pea husk, baobab shells, and marula stones. The well-correlated isotherm was the Langmuir model and the adsorbents with the best adsorption capacity were baobab shells and pigeon pea husk as compared to the others.

A chemically modified adsorbent derived from okra was explored in removing toxic metals [Pb(ii), Cd(ii), Cu(ii), and Zn(ii)] from synthetic wastewater.^104^ Batch technique tests were used to determine the impact of removal time, temperature, pH, and metal particle fixation. The experimental data conformed to Dubinin–Radushkevich and Langmuir isotherms. The values of Δ*H*_0_ and Δ*G*_0_ obtained from the thermodynamic study showed that the metals uptake on the chemically modified cellulosic biopolymer prepared from okro is unrestricted and exothermic.

Ogunleye *et al.*^105^ explored banana stalk usage in the decontamination of Pb(ii) ions from wastewater. Phosphoric acid (H_3_PO_4_) was used to modify the adsorbent at 1 : 1 (w/v). Besides, the mixture was dehydrated at 105 °C for 12 hours and further at 800 °C without air for 2 hours leading to the occurrence of carbonization. A 0.1 M HCl was applied to treat the carbonized biosorbent for 1 h. At 120 min equilibrium time, the optimum percentage removal was 97.9% however; at a pH of 8.0 and 250 mg L^−1^ Pb(ii) initial concentration, the optimum capacity attained was 200 mg g^−1^. The best kinetic model applied in analyzing the kinetic data was pseudo-second-order while the isotherm model that depicted good agreement to the equilibrium data was the Langmuir model.

Hikmat *et al.*^106^ examined Pb(ii) biosorption by absorbents obtained from agro-based materials including petiole and palm tree fiber. The uptake of Pb(ii) unto the bio-sorbents exhibited dependence on shaking time, pH, biosorbent weight, and Pb(ii) initial concentration. The isotherm data best fitted Langmuir than that of the Freundlich with a high correlation coefficient of 0.9801 and 0.9974 for fiber and petiole correspondingly. The kinetic data indicated that the pseudo-first-order fitted well with high coefficients of determination for fiber (0.9773) and petiole (0.9720), respectively.

The potential of natural bentonite and activated carbon for Pb(ii) depollution from water media was studied by Yarkandi.^107^ The study revealed that the amount of Pb(ii) removed upsurges with pH, contact time, and initial concentration but declines with adsorbent dose and temperature. The pseudo-second-order was the best fitted kinetic model while both Langmuir and Freundlich isotherms show good conformity to equilibrium data. Adsorption thermodynamic parameters suggested endothermic, spontaneous, and increase in randomness. The study concluded that bentonite is more efficient in removing Pb(ii) than activated carbon.

Vieira *et al.*^108^ conducted kinetic studies on Pb(ii) and Cu(ii) ions utilizing rice husk ash (RHA) in a fixed column bed. The study has shown that RHA could be applied as a cost-effective biosorbent for eliminating Pb(ii) and Cu(ii) ions from effluents. The optimal removal capacities attained for Pb(ii) and Cu(ii) were 0.0561 and 0.0682 mmol g^−1^ respectively at a temperature of 20 °C. Biosorption isotherm data were well-represented using Langmuir isotherm. The thermodynamic parameters evaluated showed exothermic and spontaneous adsorption of Pb(ii) whereas the adsorption system of Cu(ii) was endothermic and spontaneous.

Chandrasekaran and Jeyakumar^4^ prepared activated carbons using green *Ulva fasciata* sp. to decontaminate Pb(ii) ions from the marine. The impact in the variation of parameters such as pH, contact time, Pb(ii) concentration, and biosorbent dose was investigated. Isotherm and kinetic models have been employed and the study indicated that the Langmuir isotherm and pseudo-second-order as the best fit biosorption models.

The depollution of Pb(ii) from the water was investigated in an acidic medium (pH = 4) by Kouakou *et al.*^109^ using commercial activated carbons produced from wood and coconut shells. The study disclosed that the powdered activated carbon obtained from the wood showed a remarkably high uptake capability than the activated carbon of coconut shells. The powdered and granular activated carbons exhibited diverse sorption abilities. At 25 °C, the maximum Pb(ii) adsorbed on powdered, 830-granular, and 123-granular activated carbons were 44.58, 38.96, and 39.06 mg g^−1^, respectively. Langmuir's model showed a good representation of the isotherm data.

Hikmat *et al.*^106^ examined Pb(ii) elimination from the aqueous environment using leaf and fiber of a palm tree. The biosorption factors indicated that the removal time for Pb(ii) was a bit fast and equilibrium was accomplished at approximately 30 and 40 min by the leaf and fiber, respectively. At a pH range of 6.5–7.0, optimal Pb(ii) adsorption was attained using 0.5 g of the adsorbent. However, at high temperatures, Pb(ii) removal by the adsorbents declined to signify exothermic biosorption. The isotherm and kinetic data of Pb(ii) obtained using the two biosorbents conformed to Langmuir and pseudo-first-order than other models. It was found that the adsorption system was exothermic, feasible, and spontaneous.

Ghasemi and Gholami^110^ studied Pb(ii) elimination from synthetic water making use of Myrtaceae sawdust. The influence of pH, contact time, dosage, and initial Pb(ii) concentration was investigated using the batch technique. At a pH of 7.0, with the contact time of 30 min and 10 g L^−1^ dosage, the maximal removal efficiency of Pb(ii) was 97.49%. The biosorption of Pb(ii) upsurges with increasing initial concentration. The equilibrium data demonstrated good correspondence with the two models, Langmuir and Freundlich. The kinetic data analyzed show conformity with the second-degree kinetic equation.

Mokaddem *et al.*^111^ reported Pb(ii) sorption from aqueous media using agar beads. The impact of solution pH, biomass dosage, contact time, and initial lead(ii) concentration was examined. The study recommended agar beads for the decontamination of Pb(ii) from aqueous solutions. The experimental results have shown that biosorption equilibrium was reached below 120 min at a pH of 5.0. The Freundlich isotherm was found to show best fit the isotherm data.

Haloi and Chakravarty^112^ conducted a study to examine the ability of heartwood charcoal of *Areca catechu* (HCAC) in depolluting Pb(ii) ions from water. Batch tests were piloted to find out the biosorption characteristics at varied pH, contact times, adsorbent doses, and initial concentrations. At an equilibration time of 25 min, optimal biosorption of Pb(ii) was attained at a pH of 5.0 and 0.5 g biomass dosage. The experimental data was found to be well-fitted to the Freundlich isotherm model implying that the HCAC surface was heterogeneous while the kinetic data was better described by the pseudo-second-order model.

Athar *et al.*^113^ considered biomass obtained from *Trifolium resupinatum* in removing Pb(ii). The influences of several factors for example removal time, initial Pb(ii) concentration and pH have been explored. The study discovered that 25 min and 3.0 as optimum removal time and pH, respectively. Also, increasing the biomass dosage increased the binding aptitude of the biosorbent. The equilibrium data were analyzed with isotherm and kinetic models for which, the Langmuir and pseudo-second-order models showed the best representation of the data.

Maize husk was utilized by Adeogun *et al.*^114^ for efficient removal of Pb(ii) and Mn(ii) using a batch system. The study has shown that the elimination of Pb(ii) and Mn(ii) depended on pH, biomass dose, initial concentration, and temperature, respectively. The equilibrium data showed a good fit to the Langmuir isotherm with 7.38 and 8.52 mg g^−1^ maximum sorption capacities for Pb(ii) and Mn(ii) correspondingly on the raw biomass. However, maximum sorption capacities obtained by acid-modified biomass were 9.33 and 9.00 mg g^−1^ for Pb(ii) and Mn(ii), respectively. The adsorption thermodynamics revealed that Pb(ii) and Mn(ii) biosorption onto maize husk is feasible, spontaneous, and exothermic.

Azouaou *et al.*^115^ examined Pb(ii) removal from synthetic by using untreated orange barks. The impacts of initial pH, dosage, removal time, and initial Pb(ii) concentration were considered. It was advocated that the orange barks are efficient and economical biosorbent in eliminating Pb(ii). The study indicated that sorption equilibrium was realized in <30 min. The analysis of the equilibrium data suggested that the Langmuir isotherm correlated best.

Mitic-Stojanovic *et al.*^116^ studied Pb(ii), Cd(ii), and Zn(ii) ions decontamination from water using *Lagenaria vulgaris* shell. The study employed batch experiments and the impacts of contact time, initial pH, temperature, and stirring speed were investigated. The biosorption of the metals ions considered was rapid, attaining equilibrium after around 5 to 10 min. The biosorption showed dependence on pH with optimal pH ranging at 4.5–6.0. The influence of temperature established that the biosorption of the metal is a chemical process.

Qaiser *et al.*^117^ utilized groundnut hulls in the decontamination of Pb(ii) from wastewater. Batch experiments were piloted to determine the influence of biosorption factors including pH, initial Pb(ii) concentration, and temperature. At an optimal pH 5 ± 0.1 and 20 ± 2 °C temperature, the optimum Pb(ii) biosorption was 31.54 ± 0.63 mg g^−1^. The equilibrium data was better described by the Langmuir model with a coefficient of determination >0.98. The kinetics of biosorption followed the pseudo-second-order kinetic model.

Bhattacharyya and Sharma^118^ conducted a study to remove Pb(ii) ion from water media by well-developed *Azadirachta indica* leaves. The biosorbent gave a maximum removal of 93% with a 300 min contact time. The pseudo-second-order model was the well-fitted than the pseudo-first-order model while the two isotherms (Langmuir and Freundlich) agreed better to the experimental data with a coefficient of determination of 0.99.

Abdulkarim and Al-Rub^119^ produced activated carbon and chemically modified activated carbon from date pits to remove Pb(ii) from aqueous media. The variation of different factors was considered. The study indicated that both carbons exhibited high Pb(ii) uptake. Lead(ii) biosorption was found to rise by increasing pH and initial Pb(ii) concentration until at pH of 5.2 where maximum elimination of Pb(ii) was attained. The Langmuir and Freundlich isotherm models obeyed the experimental data using both biosorbents while the kinetic data followed the pseudo-second-order equation.

Wong *et al.*^120^ examined lead (Pb) elimination using batch experiments by tartaric acid modified rice husk. The adsorption system showed dependence on pH and demonstrated characteristics of exothermal adsorption. The pseudo-second-order model showed a good relationship with the equilibrium data than that of the pseudo-first-order model.

The adsorption potential of some agricultural adsorbents in removing Pb(ii) ions from an aqueous solution is summarized in Table 3.

**Table 3:** Adsorption potential of some agricultural bioadsorbents for Pb(ii) ions removal from aqueous solution

Adsorbent	Modifying agent	Experimental variable considered	Constants	Suited equilibrium model analysis (EMA)	EMA, *R*^2^ values	Kinetics model analysis (KMA)	KMA, *R*^2^ value	Optimum conditions	% Removal or *Q*_max_	References
Wood biomass	—	Concentration (*C*), contact time (CT), dosage (*D*)	Agitation speed, pH, temperature	Langmuir	0.986	Pseudo-second-order	0.9988	*C* = 100 mg L^−1^, CT = 4 h, *D* = 0.5 g L^−1^	100%	149
Walnut and almond shells	—	Contact time (CT), dosage (D), pH	Agitation speed, concentration, temperature	Langmuir	0.9952 and 0.9884, respectively	Pseudo-second-order	0.9902 and 0.9879, respectively	CT = 240 min, *D* = 7 mg L^−1^, pH = 6	—	150
Cotton shells	—	Contact time (CT), concentration (*C*), dosage (*D*)	pH, temperature	Freundlich	0.99	—	—	CT = 90 min, *C* = 1 mg L^−1^, *D* = 1 g L^−1^	90%	151
Pomelo leaves	—	Contact time (CT), pH	Temperature, concentration dosage	Sips	0.9769	Pseudo-second-order	0.9982	CT = 150 min, pH = 4	207.2 mg g^−1^	152
Fish scale and bean husk	—	Contact time (CT), pH, dosage (*D*), concentration (*C*), temperature (*T*)	Agitation speed	Temkin and Langmuir, respectively	0.5178 and 0.8978, respectively	Pseudo-second-order	1.000 and 0.9999, respectively	CT = 30 min, pH = 7, *D* = 0.2 and 6 g, respectively, *C* = 60 and 80 mg L^−1^, respectively and *T* = 321 and 318 K, respectively	90%	153
Coconut waste	HCl	pH, agitation time (AT), concentration (*C*)	Agitation speed dosage, temperature	Langmuir	0.969	Pseudo-second-order	0.999	pH = 6, *T* = 1440 min, *C* = 125 mg L^−1^	50.33 mg g^−1^	154
Winemaking waste	—	Stirring rate (SR), temperature (*T*), pH, dosage (*D*)	Contact time, temperature	Langmuir	0.9989	Pseudo-second-order	0.9999	SR = 500–750 rpm, *T* = 288 K, pH = 4, *D* = 0.25 g L^−1^	58 mg g^−1^	155
Lobeira fruit	—	pH, dosage (*D*), contact time (CT)	Agitation speed, concentration, temperature	Langmuir	0.9611	—	—	pH = 2, *D* = 250 mg, CT = 60 min	93%	156
Eggplant peel	—	Contact time (CT), pH, dosage (*D*) concentration (*C*), and temperature (*T*)	Agitation speed	Langmuir	0.9996	Pseudo-second-order	0.9998	CT = 60 min, pH = 4, *C* = 70 ppm, *D* = 0.01 g mL^−1^, *T* = 25 °C	88.33 mg g^−1^	157
Shrimp shells chitosan	NaOH	Contact time (CT), pH	Temperature, concentration dosage	—	—	—	—	CT = 90 min, pH = 4	99.88%	158
Sugarcane bagasse	—	Concentration (*C*), dosage (*D*), contact time (CT)	Agitation speed, pH, temperature	Freundlich	0.999	—	—	*C* = 7 ppm, *D* = 1 g, CT = 15 min	90–98%	159
Coffee husk	—	Dosage (*D*), contact time (CT), and concentration (*C*)	Agitation speed, pH, temperature	Freundlich	0.9800	Pseudo-second-order	1.000	*D* = 2 g L^−1^, CT = 60, *C* = 400 mg L^−1^	98%	160
Cucumber peel	—	pH, contact time (CT), and temperature (*T*)	Agitation speed, dosage, concentration	Langmuir	0.999	Pseudo-second-order	1.000	pH = 5, CT = 60 min, *T* = 20–35 °C	90%	161
Black walnut husk	—	pH, contact time (CT), concentration (*C*), dosage, and temperature (*T*)	Agitation speed	Freundlich	0.964	Pseudo-second-order	0.999	pH = 4, CT = 60 min, *D* = 0.5 g L^−1^, *T* = 308–343 K	96%	162
Sugarcane bagasse derived activated carbon	H_2_SO_4_	pH, contact time (CT), concentration (*C*)	Agitation speed, temperature, dosage	Langmuir	0.9508	Pseudo-second-order	0.9942	pH = 5, CT = 180 min, *C* = 50 mg L^−1^	23.4 mg g^−1^	163
*Spirulina platensis* biomass	—	pH, dosage (*D*), temperature (*T*), concentration (*C*), and contact time (CT)	Agitation speed	Freundlich	0.9700	—	—	pH = 3, *D* = 2 g L^−1^, *T* = 26 °C, *C* = 100 mg L^−1^, CT = 60 min	>91%	164
Pineapple waste	NaOH	pH, contact time (CT), temperature (*T*)	Agitation speed, dosage, concentration	—	—	—	—	pH = 4, CT = 30 min, *T* = 60 °C	>95%	165

### 2.6 Adsorption kinetic studies

Adsorption kinetic modeling is conducted to determine the rate of biosorption and expressions for a certain reaction. Adsorption kinetics are significant in the treatment of wastewater as it provides valued information on the reaction pathways and in the mechanism of the adsorption process.^114^ The adsorption kinetics has key parameters for describing the basic properties of a good adsorbent. It offers cherished information about the system and regulates the adsorbate reactions in the solid–solute interface at different times.^1,91^ Many kinetic models had been applied in the elimination of pollutants in water and wastewater including pseudo-first-order, pseudo-second-order, Elovich, and intraparticle diffusion models.

For the present review, the linearized adsorption kinetic models commonly utilized in the elimination of Pb(ii) from water and wastewater are presented in Table 4.

**Table 4:** Linearized adsorption kinetics model equations

Model	Linear form	Plot	References
Pseudo-first-order	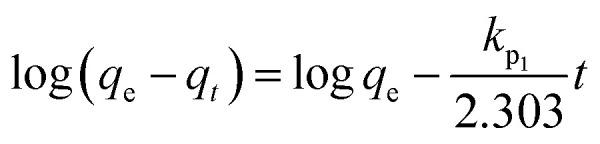	log(*q*_e_ − *q*_*t*_) *vs. t*	2, 3 and 166
Pseudo-second order	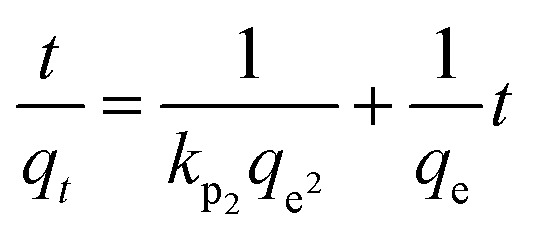	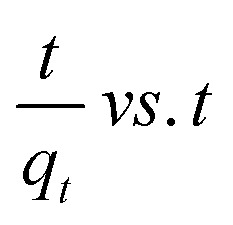	2, 76 and 167
Elovich	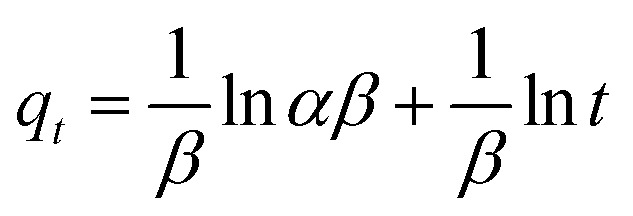	*q*_*t*_ *vs.* ln *t*	89, 168 and 169
Intra-particle diffusion	*q*_*t*_ = *k*_id_*t*^1/2^ + *C*	*q*_*t*_ *vs. t* ^1/2^	4, 168 and 170

### 2.7 Adsorption isotherm studies

Adsorption isotherms are investigated to describe the adsorption system. Adsorption isotherm models signify the link between the concentration of adsorbate adsorbed on the biosorbent and the concentration of adsorbate retained in the adsorption medium.^86,113,121^

Many isotherm models are frequently used to fit the adsorption equilibrium data to get a linear regression for the prediction of the maximal sorption capacity of the employed adsorbent.^91^ In most of the numerous studies reviewed, the adsorption processes were justified by applying linear forms of one-parameter adsorption isotherm model (Henry), two-parameter adsorption isotherm models (Langmuir, Freundlich, Dubinin–Radushkevich, Temkin, Harkin–Jura, and Elovich), and three-parameter adsorption isotherm models (Redlich–Peterson and Jossens) as summarized in Table 5.

**Table 5:** Linearized adsorption isotherm model equations

Isotherm	Linear form	Plot	Significance	References
Henry	*q*_e_ = *K*_HE_*C*_e_	*q*_e_ = *K*_HE_*C*_e_	For very low concentrations	2 and 86
Langmuir	(I) 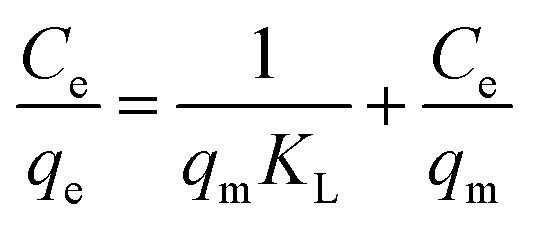	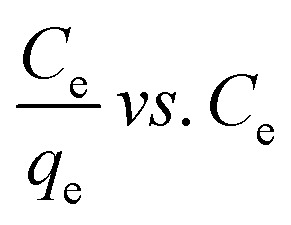	For monolayer adsorption on homogeneous surfaces	2, 86, 121 and 171
	(II) 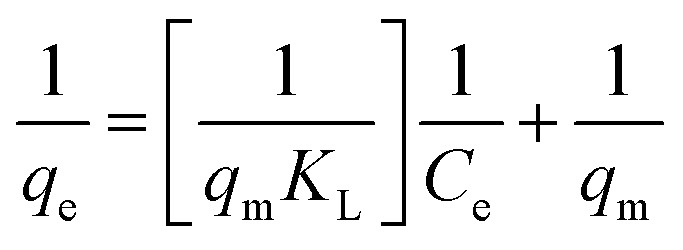	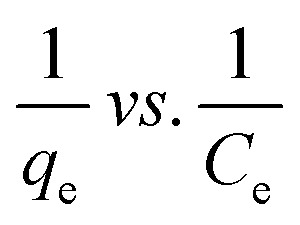		
	(III) 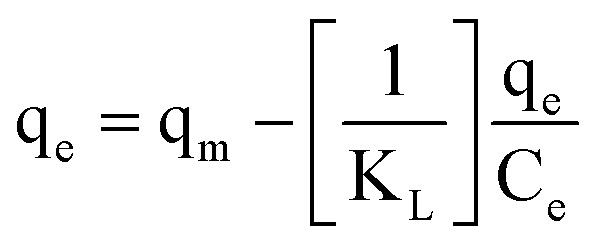	*q*_e_ *vs. C*_e_		
	(IV) 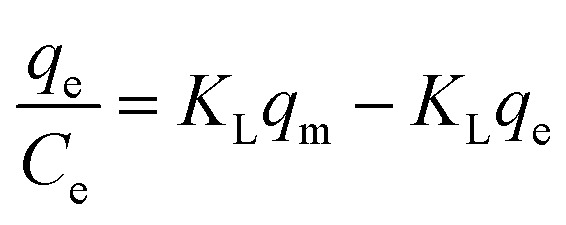	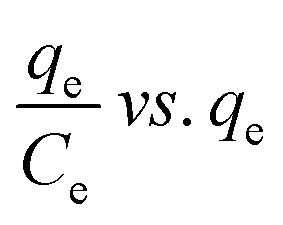		
Freundlich	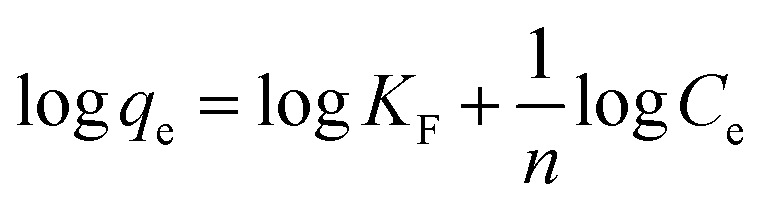	log *q*_e_*vs.* log *C*_e_	For dilute solutions, over a small concentration range	2, 86, 121 and 172
Dubinin–Radushkevich (D–R)	ln q_e_ = ln *q*_s_ − *K*_D_*ε*^2^	ln *q*_e_*vs. ε*^2^	Describes adsorption with a Gaussian energy distribution onto a heterogeneous surface	2, 86 and 173
	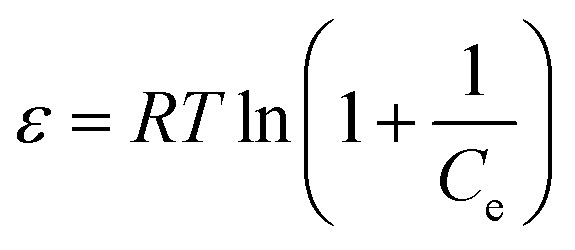			
Temkin	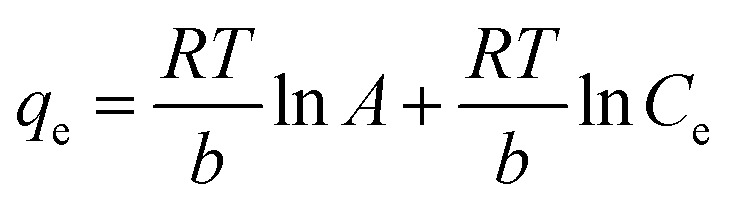	*q*_e_ *vs.* ln(*C*_e_)	Based on the adsorbent–adsorbate interactions	2, 86 and 174
	or *q*_e_ = *B* ln *A* + *B* ln *C*_e_			
	Where 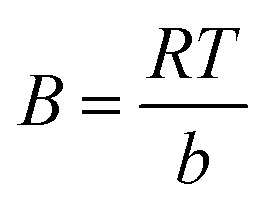			
Harkin–Jura	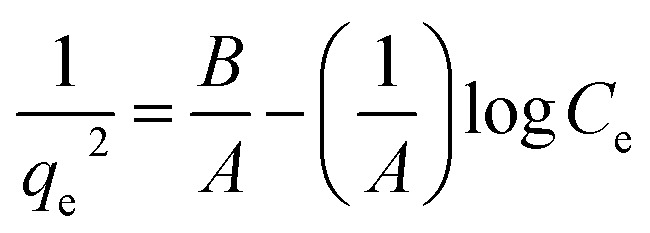	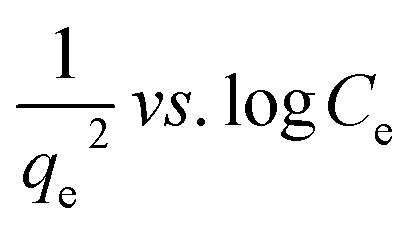	For multilayer adsorption on the absorbent surface with heterogeneous pore distribution	86 and 175
Elovich	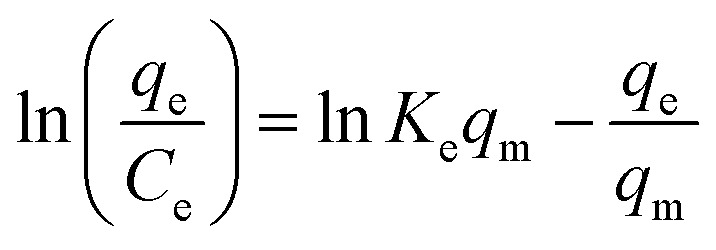	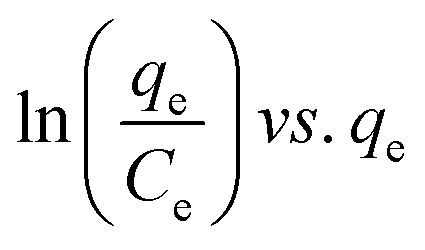	Based on chemical adsorption kinetics of adsorbate unto biomaterials	86 and 176
Redlich–Peterson (R–P)	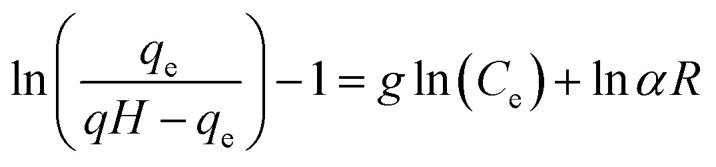	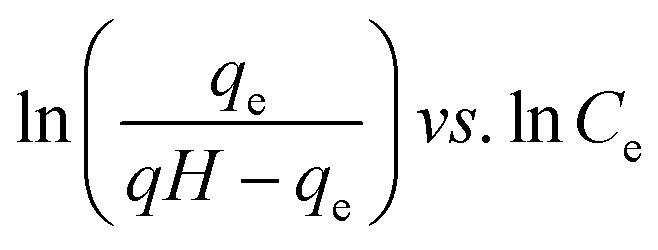	It can be applied either in homogeneous or heterogeneous systems	2, 86, 177 and 178
Jossens	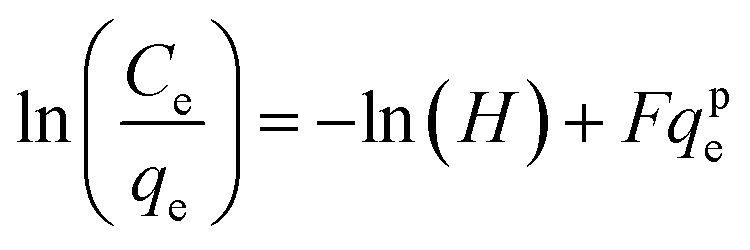	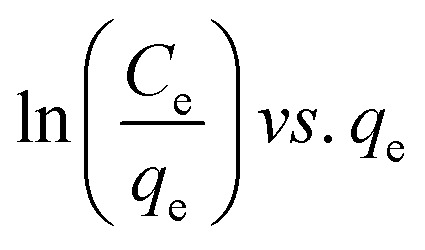	For energy dissemination of solute–solid interactions	86, 177 and 179

## 3. Conclusions

The review of the various studies on Pb(ii) biosorption indicates that low-cost biosorbents are eco-friendly, cost-effective, and simple techniques for water and wastewater treatment containing lead(ii) ions. Pb(ii) biosorption with these low-cost biosorbents was found to show dependence on several adsorption factors such as pH, contact time, adsorbent dose, initial Pb(ii) concentration, and temperature. Furthermore, the efficiency of the biosorption procedures was subjected to physicochemical characteristics of the biomass used as the biosorbent. In most of the studies, batch adsorption data have been modeled by determining the kinetic, isothermal, and thermodynamic parameters to elucidate the mechanism of the adsorption system. Most of the studies on the adsorptive removal of Pb(ii) were found to follow the pseudo-second kinetic and Langmuir isotherm models with the thermodynamics variables suggesting the feasibility and spontaneous nature of Pb(ii) sequestration. However, gaps exist to increase biosorption ability, economic feasibility, optimization of the biosorption system, and desorption and regeneration of the used agricultural biosorbents.

## 4. Future perspectives

Various laboratory-scale investigations have demonstrated that the capability of unconventional biosorbents in terms of effectiveness for the removal of Pb(ii) from aqueous systems is extremely high. However, because of the lack of confidence in the engineering of these materials and practicality, they are not utilized on an industrial scale. Therefore, it is critical to reconsider multidimensional and multidisciplinary research approaches in biosorbent production for commercial and industrial applications in the near future to minimize metal burdens in aquatic systems.

Another consideration is that industrial effluents do not contain a single contaminant, but rather a large mixture of heterogeneous contaminants that may have a larger influence on adsorption systems and selectivity. As a result, significant measures should be taken to develop and apply novel solid materials, such as biomass, synthetic nanomaterials, and chitosan as low-cost biosorbents, for the decontamination of various toxic heavy metals from water systems. Therefore, deeper studies into the adsorption mechanisms are required, with a particular emphasis on the simultaneous detoxification of multiple heavy metals ions using cheap and single adsorbents. Besides, future research should look into different combinations of agro-industrial wastes and by-products that can effectively remove heavy metal ions from aqueous media.

The literature review also uncovered a current trend in this area of study, with a significant shift toward adopting more sustainable techniques and new technologies that prioritize sustainability over relying solely on the efficiency and performance of the technology in treating wastewater containing heavy metals ions. Finding the right balance among heavy metals decontamination and recovery would be one of those sustainability issues. As a result of this development, the attention and motivation have shifted away from simple wastewater treatment to the retrieval and recycling of heavy metals as well as the spent biosorbents for further use. Therefore, to avoid further environmental pollution, other feasible alternatives and dependable procedures should be employed for heavy metals recovery, regeneration of exhausted biosorbents, and safe disposal of spent biosorbents.

## Author contributions

Jonas Bayuo: conceptualization, investigation and writing – original draft. Mwemezi Rwiza: reviewing, editing, and supervision. Kelvin Mtei: editing and supervision.

## Conflicts of interest

The authors declare that there is no conflict of interest.

## Supplementary Material
